# Methotrexate-mediated activation of an AMPK-CREB-dependent pathway: a novel mechanism for vascular protection in chronic systemic inflammation

**DOI:** 10.1136/annrheumdis-2014-206305

**Published:** 2014-12-30

**Authors:** C C Thornton, F Al-Rashed, D Calay, G M Birdsey, A Bauer, H Mylroie, B J Morley, A M Randi, D O Haskard, J J Boyle, J C Mason

**Affiliations:** 1 Vascular Sciences, Imperial Centre for Translational and Experimental Medicine, National Heart and Lung Institute, Imperial College London, Hammersmith Hospital, London, UK; 2 King Fahad Cardiac Centre, King Saud University, Riyadh, Saudi Arabia; 3 University of Bath, Bath, UK

**Keywords:** Cardiovascular Disease, Inflammation, Methotrexate, Rheumatoid Arthritis, Atherosclerosis

## Abstract

**Aims:**

Premature cardiovascular events complicate chronic inflammatory conditions. Low-dose weekly methotrexate (MTX), the most widely used disease-modifying drug for rheumatoid arthritis (RA), reduces disease-associated cardiovascular mortality. MTX increases intracellular accumulation of adenosine monophosphate (AMP) and 5-aminoimidazole-4-carboxamide ribonucleotide which activates AMP-activated protein kinase (AMPK). We hypothesised that MTX specifically protects the vascular endothelium against inflammatory injury via induction of AMPK-regulated protective genes.

**Methods/results:**

In the (NZW×BXSB)F_1_ murine model of inflammatory vasculopathy, MTX 1 mg/kg/week significantly reduced intramyocardial vasculopathy and attenuated end-organ damage. Studies of human umbilical vein endothelial cells (HUVEC) and arterial endothelial cells (HAEC) showed that therapeutically relevant concentrations of MTX phosphorylate AMPKα^Thr172^, and induce cytoprotective genes including manganese superoxide dismutase (MnSOD) and haem oxygenase-1 (HO-1). These responses were preserved when HUVECs were pretreated with tumour necrosis factor-α to mimic dysfunctional endothelium. Furthermore, MTX protected against glucose deprivation-induced endothelial apoptosis. Mechanistically, MTX treatment led to cyclic AMP response element-binding protein (CREB)^Ser133^ phosphorylation, while AMPK depletion attenuated this response and the induction of MnSOD and HO-1. CREB siRNA inhibited upregulation of both cytoprotective genes by MTX, while chromatin immunoprecipitation demonstrated CREB binding to the MnSOD promoter in MTX-treated EC. Likewise, treatment of (NZW×BXSB)F_1_ mice with MTX enhanced AMPKα^Thr172^ phosphorylation and MnSOD, and reduced aortic intercellular adhesion molecule-1 expression.

**Conclusions:**

These data suggest that MTX therapeutically conditions vascular endothelium via activation of AMPK-CREB. We propose that this mechanism contributes to the protection against cardiovascular events seen in patients with RA treated with MTX.

## Introduction

Premature cardiovascular disease (CVD) is a serious long-term complication of chronic systemic inflammatory diseases including rheumatoid arthritis (RA) and systemic lupus erythematosus (SLE). Despite advances in disease-modifying and biological therapy for these diseases, we lack specific strategies aimed at retarding development of premature CVD and have limited knowledge of whether individual drugs offer vascular protection. Since endothelial dysfunction precedes overt atherosclerosis, investigating the ability of drugs to activate cytoprotective signalling pathways that prevent or reverse endothelial dysfunction is an attractive research strategy. Cytoprotective pathways include those regulated by AMP-activated kinase (AMPK). AMPK, a ubiquitous signalling kinase composed of a heterotrimeric complex of a catalytic α subunit and regulatory β and γ subunits, is generally considered a sensor of cellular metabolic status.^1^ In endothelial cells (EC), AMPK activity exerts multiple protective effects including enhanced endothelial nitric oxide synthase phosphorylation and nitric oxide synthesis,^2^ mitochondrial biogenesis,^3^ and protection against apoptosis^4^ and oxidative damage.^5^ ^6^

Methotrexate (MTX) therapy is central to the current treatment paradigms for RA. There is sound evidence that MTX reduces CVD in RA^7^ ^8^ and improves clinical markers of endothelial dysfunction.^9^ These findings led to the recently initiated Cardiovascular Inflammation Reduction Trial, in which MTX or placebo is prescribed to patients with prior myocardial infarction (MI) to test the inflammatory hypothesis of atherothrombosis:^10^ an intriguing new direction in the treatment of CVD.

Mechanistic understanding of the vasculoprotective actions of MTX is sparse. However, it is known that long-lasting polyglutamate metabolites of MTX inhibit 5-aminoimidazole-4-carboxamide ribonucleotide (AICAR) transformylase and adenosine deaminase, leading to a rise in intracellular concentrations of AICAR-monophosphate (ZMP) and AMP.^11^ The subsequent accumulation and extracellular release of adenosine has been proposed as the principal anti-inflammatory mechanism of action of MTX.^12^ ^13^ However, both ZMP and AMP are AMPK activators. We, therefore, hypothesised that the low-dose MTX regimen used in contemporary rheumatology practice activates AMPK, leading to enhanced expression of cytoprotective proteins within vascular EC: a new mechanism of action to explain its clinical action in reducing CVD associated with inflammation.

## Materials and methods

Detailed methods are supplied in the online supplementary file.

(NZW×BXSB)F1 (WBF1) male mice were bred from female New Zealand White (Harlan, UK) and male BXSB mice (bred in-house). They were studied according to UK Home Office guidelines and with ethical approval from Imperial College (Licence PPL 70/6722). Animals were treated from 12 to 16 weeks with MTX 1 mg/kg (TEVA, UK) or an equal volume of 0.9% saline by weekly intraperitoneal injection. After euthanasia, the heart was paraffin-embedded and sections stained with periodic acid-Schiff (PAS) or picrosirius red for analysis of the vascular disease or myocardial infarct size. The aorta was snap-frozen, sectioned transversely or ground and analysed by immunofluorescence or immunoblotting.

Human umbilical vein EC (HUVEC) and human aortic EC (HAEC) were treated for up to 72 h with MTX 0.1–100 nM before analysis by immunoblotting, quantitative real-time PCR, flow cytometry and chromatin immunoprecipitation.

## Results

### MTX reduces the severity of intramyocardial vasculopathy and attenuates organ damage in WBF1 mice

To demonstrate the vasculoprotective properties of low-dose MTX in vivo, (NZW×BXSB)F1 (WBF1) mice were studied. WBF1 mice develop SLE, characterised by glomerulonephritis, anti-DNA and antiphospholipid antibodies.^14^ ^15^ A high proportion (up to 80%) develop vasculopathy of the muscular intramyocardial arteries.^16^ Pathological features include an adventitial inflammatory cell infiltrate, deposition of PAS-positive immunoglobulin and complement within the arterial wall, and progression to arterial occlusion and MI.^17^

WBF1 mice were treated with MTX 1 mg/kg by weekly intraperitoneal injection for 4 weeks from 12 to 16 weeks of age. The dose of MTX chosen is representative of long-term low-dose MTX therapy used to treat inflammatory diseases clinically, and falls between those concentrations shown to increase adenosine levels and protect against collagen-induced arthritis.^13^ ^18^ At 12 weeks, disease is detectable in WBF1 mice,^16^ thus, intervention was timed to model the treatment of early systemic inflammatory disease in patients and to demonstrate reversibility of the vasculopathy.

**Figure 1:**
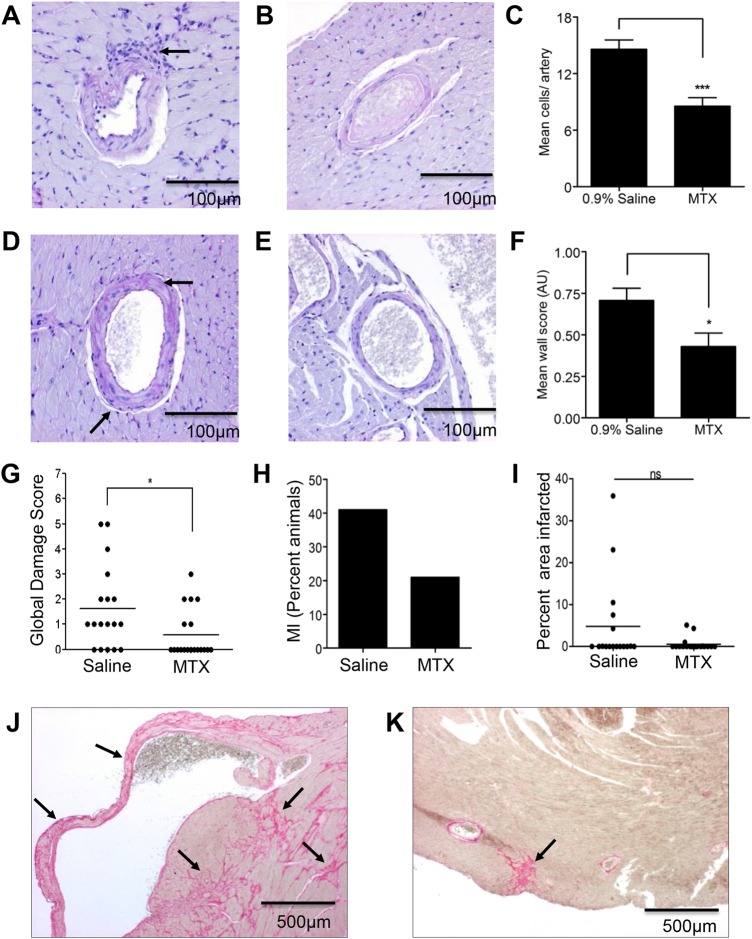
Methotrexate (MTX) attenuates intramyocardial vasculopathy and reduces severity of organ damage in WBF1 mice. Male WBF1 mice were treated with MTX 1 mg/kg (n=19) or an equal volume of 0.9% saline (n=18) by intraperitoneal injection weekly for 4 weeks from 12 to 16 weeks of age. Short-axis paraffin-embedded sections of heart were stained with periodic acid-Schiff (PAS) and picrosirius red. Vasculopathy was quantified by counting cells infiltrating the adventitia of intramyocardial arteries and by scoring PAS-positive staining in the vessel walls, and myocardial infarction (MI) area was quantified by picrosirius red staining. A disease-related damage score (see online supplementary table) was constructed based on findings at postmortem. (A) An artery with a periadventitial leucocytic infiltrate (arrow), compared with normal artery (B) and (C) quantification of the mean number of adventitial cells per vessel. (D) Artery with severe thickening of the PAS-positive basement membrane (arrow) and compared with normal artery (E) and (F) quantification of the arterial vasculopathy score shows (G) the global damage score, (H) the incidence of MI, and (I) quantification of the total area of infarcted myocardium, quantified by picrosirius red staining of short-axis sections of heart. (J) and (K) Representative photomicrographs of picrosirius red staining with (J) extensive fibrotic infarcts involving the right ventricle and septum (arrows) in a saline-treated animal, and (K) a small fibrotic infarct in the left ventricle epicardium of a MTX-treated animal (arrow). ns, not statistically significant; *p<0.05; ***p<0.001.

Following 4 weeks treatment with MTX, a significant reduction in the intramyocardial vasculopathy was observed. This was quantified by first counting infiltrating leucocytes in the adventitia of each intramyocardial artery in a single section per animal (figure 1A–C); and second, by scoring the deposition of PAS-positive material in arterial walls (figure 1D–F).

Furthermore, MTX reduced end-organ damage in WBF1 mice. A multisystem organ damage score (see online supplementary table) used postmortem demonstrated a significant protective effect of MTX (figure 1G). Specifically relating to cardiac disease, at 16 weeks, 41% of untreated mice in our cohort had an MI consistent with previous reports in this model at this time point (figure 1H).^16^ ^19^ Although the optimal time to identify an effect of MTX on infarct incidence would be at 24–30 weeks, when the frequency of MI is 53%–62%, data would be confounded by the onset of disease-related renal impairment and consequent increased MTX toxicity. Thus, although not reaching statistical significance at the earlier 16-week time-point, fewer MTX-treated mice suffered an MI (21% vs 41% in the untreated animals), with infarcts smaller in the treated mice (figure 1H–K).

Thus, low-dose MTX reduces inflammatory vascular injury in vivo, leading to a reduction in end-organ damage in WBF1 mice.

### MTX phosphorylates AMPK and induces MnSOD and other cytoprotective target genes

**Figure 2:**
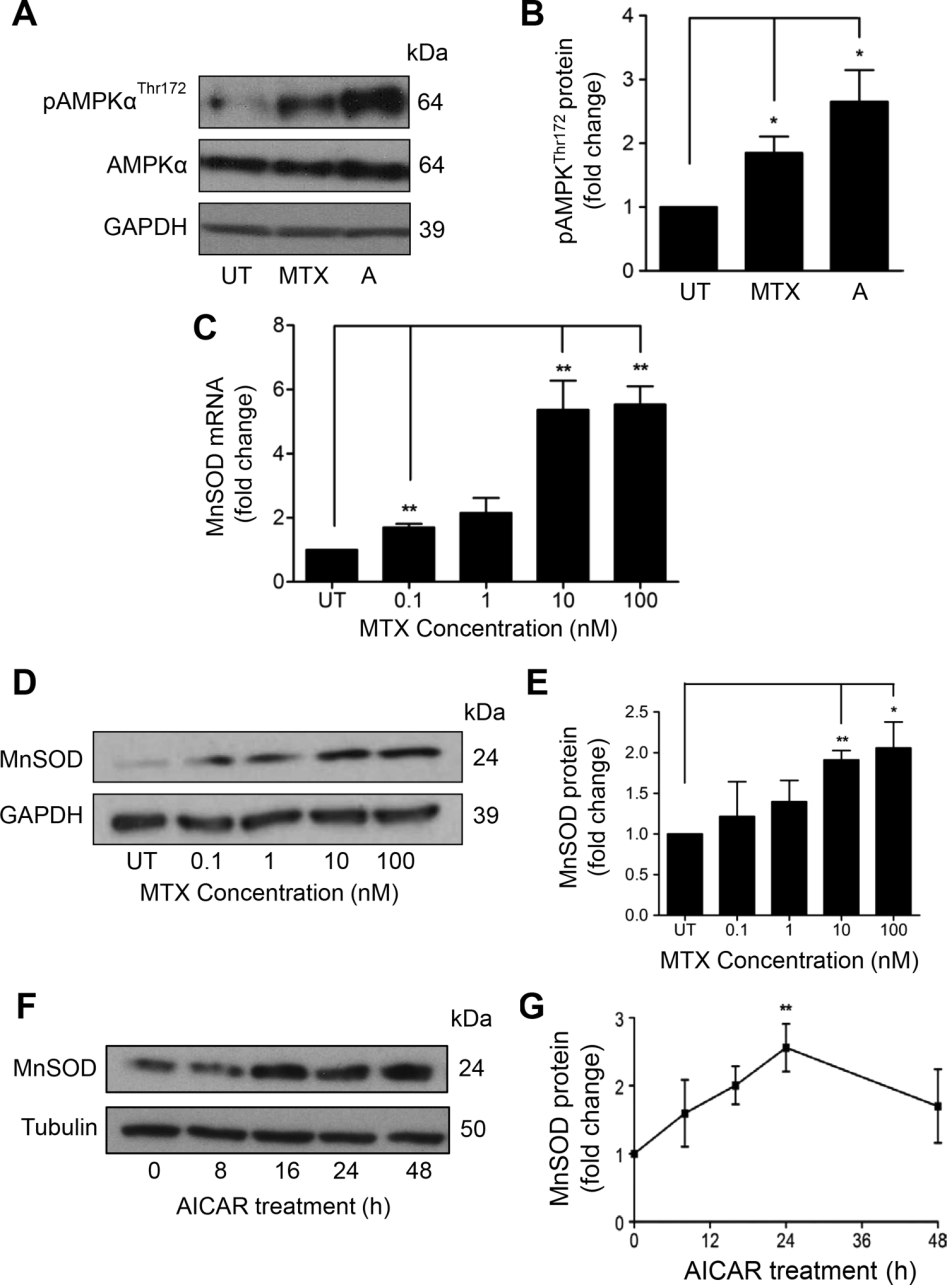
MTX treatment leads to AMPKα phosphorylation and induction of MnSOD mRNA and protein. (A) and (B) HUVEC were treated with MTX 100 nM for 48 h. AMPKα phosphorylation was demonstrated by immunoblot (A; with densitometry B), using AMPK agonist AICAR 1 mM as positive control. HUVECs were treated with MTX 0–100 nM and (C) MnSOD mRNA, and (D) and (E) MnSOD protein were quantified after 48 h by qRT-PCR and immunoblotting, respectively. (F) and (G) HUVEC were treated with AICAR for up to 48 h and MnSOD quantified by immunoblotting. Each experiment was performed three to five times. A: AICAR, 5-aminoimidazole-4-carboxamide ribonucleotide; AMPK, AMP-activated protein kinase; GAPDH, glyceraldehyde-3-phosphate dehydrogenase; HUVEC, human umbilical vein endothelial cells; MnSOD, manganese superoxide dismutase; MTX, methotrexate; UT, untreated. *p<0.05; **p<0.01.

To identify potential mechanisms underpinning vascular protection, we investigated the hypothesis that, through its effects on nucleotide metabolism, MTX may activate AMPK, a signalling kinase with significant vascular protective actions, including manganese superoxide dismutase (MnSOD) induction.^20^ MnSOD is a mitochondrial antioxidant enzyme that catalyses the conversion of superoxide into hydrogen peroxide. Induction of MnSOD is an important vascular cytoprotective response, capable of protecting against mitochondrial damage and atherogenesis.^21^ To establish whether MTX activates human endothelial AMPK, HUVECs were exposed to 100 nM MTX for up to 72 h. The concentration chosen is able to increase intracellular AICAR in vitro^12^ and is achievable in patient plasma following conventional low-dose therapeutic dosing.^22^ AMPKα^Thr172^ phosphorylation was observed after 48 h treatment, but not earlier, in HUVEC (figure 2A, B; 24 and 72 h data not shown) and in HAECs (see online supplementary figure IA, B). The delay in phosphorylation suggests an indirect action, such as the accumulation of AICAR and AMP following inhibition of AICAR transformylase by MTX polyglutamate metabolites, rather than a direct action of MTX on upstream AMPK-kinases.

**Figure 3:**
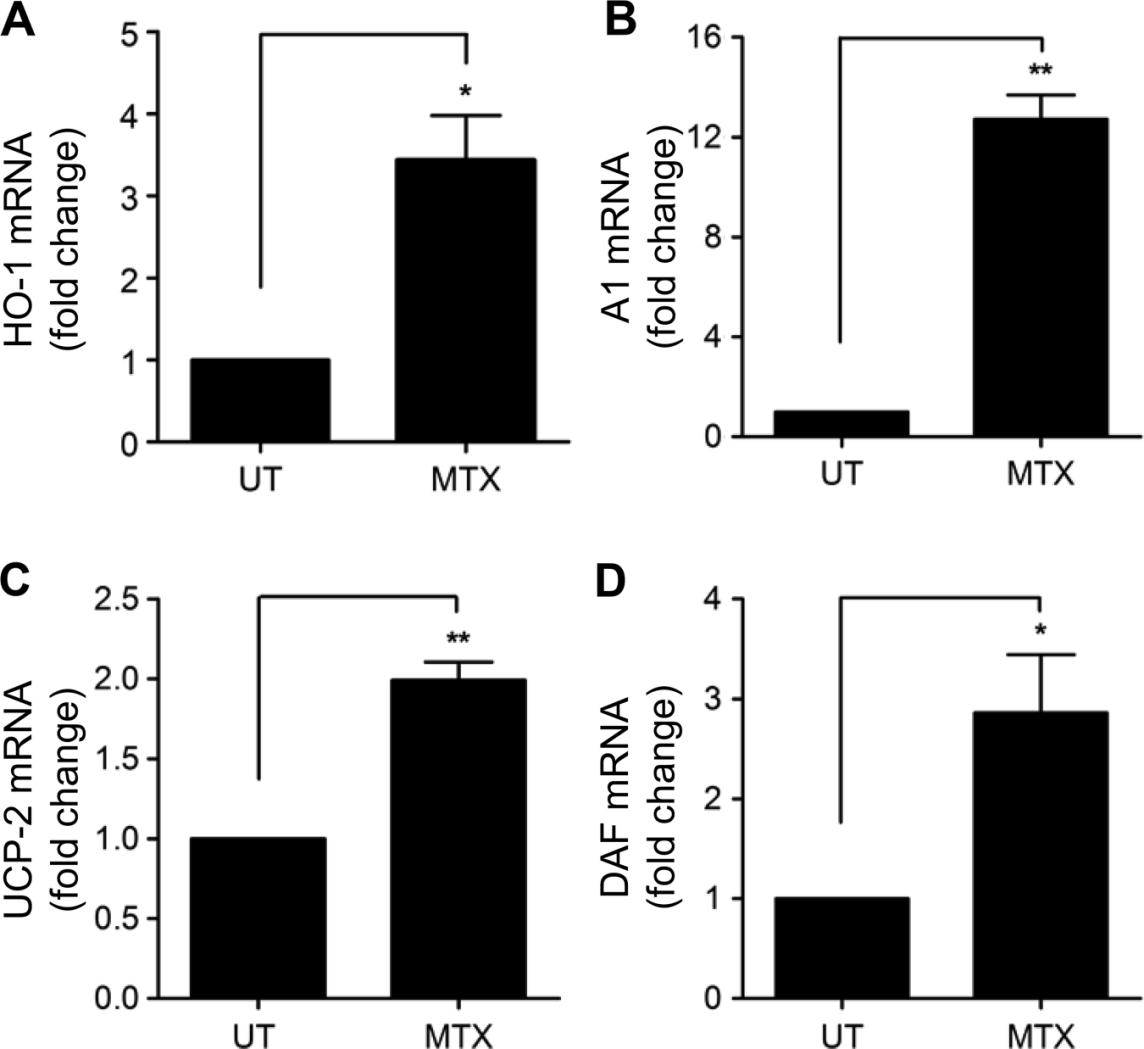
MTX treatment results in the induction of AMPK target genes. HUVEC were treated with MTX 100 nM for 48 h and mRNA levels of (A) HO-1, (B) A1, and (C) UCP-2 mRNA were analysed by qRT-PCR. (D) Induction of DAF mRNA was measured after 72 h MTX treatment. Each graph represents the results of four experiments. AMPK, AMP-activated protein kinase; A1, Bcl-2-related protein A1; DAF, decay-accelerating factor; HO-1, haem oxygenase-1; HUVEC, human umbilical vein endothelial cells; MTX, methotrexate; UCP-2, uncoupling protein-2; UT, untreated. *p<0.05; **p<0.01.

In addition to AMPK phosphorylation, induction of MnSOD mRNA and protein was seen after 48 h of MTX treatment. This response was concentration-dependent, first seen with MTX 0.1 nM and maximal at 10 and 100 nM (see figure 2C–E; time course online supplementary figure IC) and reproducible in HAECs (see online supplementary figure ID, E). Increased MnSOD was also detected following AICAR treatment of HUVECs (figure 2F, G), and has previously been linked with AMPK activity.^3^ ^6^ ^23^ In addition to MnSOD, 48 h exposure to low-dose MTX increased expression of other important endothelial cytoprotective genes. These included the antioxidant, anti-inflammatory, antiapoptotic enzyme haem oxygenase-1 (HO-1; figure 3A and online supplementary figure IF, G); Bcl-2-related protein Al (A1), an antiapoptotic member of the Bcl-2 family (figure 3B), and uncoupling protein-2 (UCP2), important for regulation of mitochondrial-reactive oxygen species generation (figure 3C). After 72 h, induction of the complement-inhibitory protein decay-accelerating factor (DAF, CD55) was also observed (figure 3D). Thus, MTX-mediated AMPK activation in EC is linked to the regulation of a variety of AMPK-dependent protective genes, all of which are antiatherogenic.

### MnSOD induction by MTX requires AMPK and CREB activation

**Figure 4:**
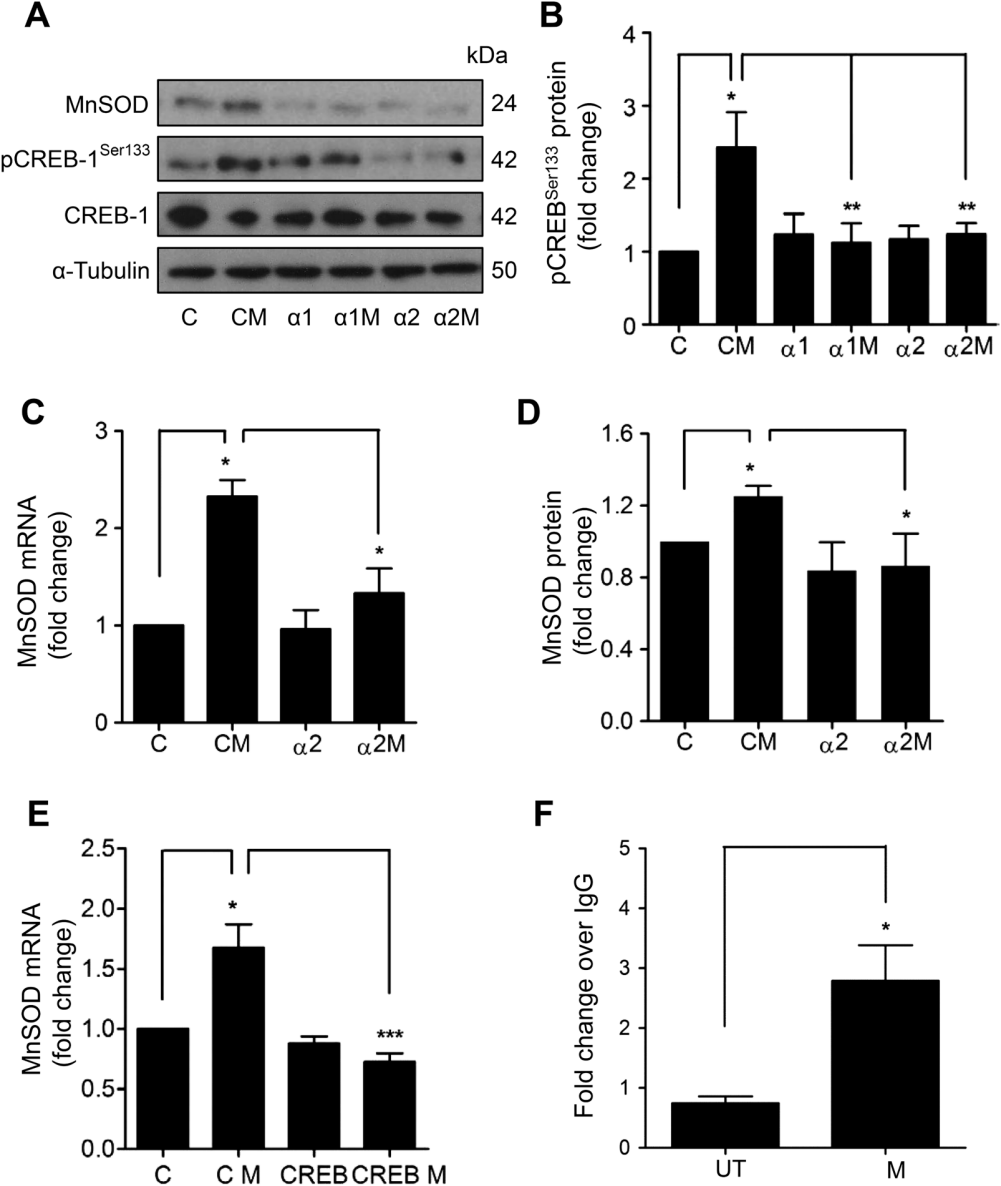
Methotrexate (MTX) activates an AMP-activated protein kinase (AMPK) and cyclic AMP-response element binding protein (CREB) linear signalling pathway to induce manganese superoxide dismutase (MnSOD). (A–D) AMPKα1 or AMPKα2 subunits were depleted in human umbilical vein endothelial cells (HUVEC) by RNA interference, prior to addition of MTX (100 nM) for 48 h and analysis of: (A) MnSOD, CREB^Ser133^ phosphorylation and total CREB by immunoblotting. (B) Densitometric quantification of changes in CREB phosphorylation. Changes in (C) MnSOD mRNA and (D) MnSOD protein. (E) HUVECs transfected with control or CREB siRNA were treated with MTX and MnSOD mRNA analysed by qRT-PCR. (F) HUVECs were treated with MTX for 48 h for chromatin immunoprecipitation analysis of CREB binding of the MnSOD promoter. Following immunoprecipitation with an anti-CREB monoclonal antibody or negative control immunoglobulin G (IgG), isolated chromatin was used in a qRT-PCR reaction with primers spanning CREB binding sites within the MnSOD promoter and a downstream control site. Data is presented normalised to input DNA and IgG control. α1, AMPKα1 siRNA; α1M, AMPKα1 siRNA and MTX; α2, AMPKα2 siRNA; α2M, AMPKα2 siRNA and MTX; C, scrambled control siRNA; CM, control siRNA and MTX; CREB, CREB siRNA; CREB M, CREB siRNA and MTX; UT, untreated. *p<0.05; **p<0.01; ***p<0.001.

To dissect further, the transcriptional pathway involved in MnSOD induction by MTX, cyclic AMP-response element binding protein (CREB) was identified as a potential candidate. CREB is a direct downstream target of AMPK,^23^ ^24^ its activity is positively associated with vascular health,^25^ and it has been implicated in MnSOD induction.^26^ CREB^Ser133^ phosphorylation was observed following treatment of HUVEC with MTX (figure 4A, B). In order to determine the role of AMPK, a loss-of-function approach was adopted. HUVECs were transfected with siRNA directed against the AMPKα1 or α2 subunit (see online supplementary figure IIA, B), prior to MTX treatment. MTX-mediated CREB^Ser133^ phosphorylation was abrogated by AMPKα1 or α2 siRNA (figure 4A, B), suggesting a linear signalling pathway between AMPK and CREB.

Next, the same approach was used to establish whether MnSOD induction by MTX is dependent upon AMPK activation. Induction of MnSOD mRNA and protein by MTX was significantly attenuated by AMPKα2 siRNA (figure 4A, C, D). Similar results were obtained following depletion of AMPKα1 (figure 4A; quantification data not shown). Likewise, analysis of HO-1 expression showed that depletion of AMPKα1 or α2 inhibited MTX-mediated HO-1 induction (see online supplementary figure IIC–E). Additionally, treatment of HUVECs with Compound C, a pharmacological AMPK antagonist, significantly reduced induction of MnSOD and HO-1 mRNA by MTX (see online supplementary figure IIF, G). Further investigation demonstrated the functional role of CREB, with siRNA depletion (see online supplementary figure IIIA, B) preventing MnSOD and HO-1 induction by MTX (see figure 4E and online supplementary figure IIIC).

Chromatin immunoprecipitation was performed to look for enhanced CREB binding to the MnSOD promoter in HUVECs treated with MTX. Analysis of the *SOD2* promoter (GenBank accession number AF059197) using MatInspector software identified two strong potential CREB binding sites (see online supplementary figure IV), the more proximal of which has previously been validated using a reporter assay and a series of deletion constructs.^26^ Significant enrichment of CREB binding to the MnSOD promoter following MTX treatment was found using primers designed to interrogate the known validated binding site (figure 4F). No enhanced binding was seen with primers designed around a negative control downstream region.

### MnSOD and HO-1 induction by MTX is maintained when ECs are treated with folic acid or TNFα

To determine whether the low-dose MTX-induced changes in gene expression in quiescent ECs are relevant clinically, we investigated responses in cells coadministered with folic acid (FA), and those exposed to tumour necrosis factor α (TNFα) to model an activated, dysfunctional endothelium.

**Figure 5:**
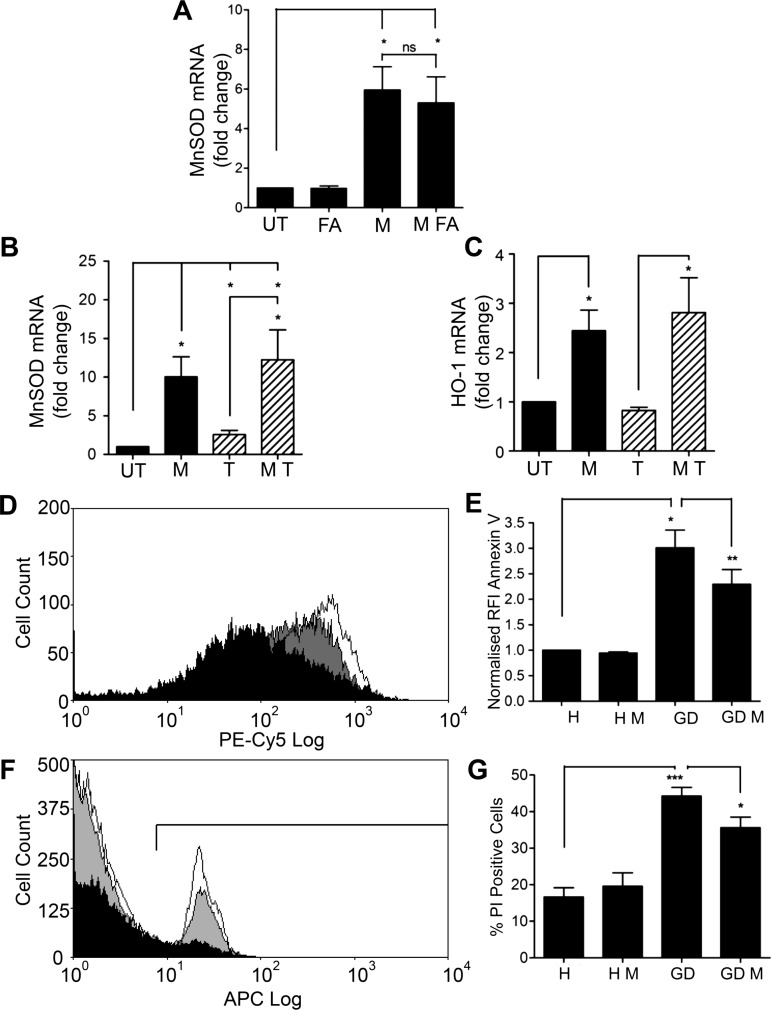
Manganese superoxide dismutase (MnSOD) induction by methotrexate (MTX) is not altered by cotreatment with folic acid (FA) or by pre-treatment with tumour necrosis factor α (TNFα), and MTX protects endothelial cells (EC) against apoptosis induced by glucose deprivation. (A) Human umbilical vein EC (HUVEC) were treated with MTX 10 nM in the presence and absence of FA 50 nM for 48 h. MnSOD mRNA was quantified by qRT-PCR. (B) and (C) HUVECs were pretreated with TNFα 1 ng/mL for 24 h prior to MTX 10 nM for 48 h. Changes in (B) MnSOD mRNA, and (C) haem oxygenase-1 (HO-1) mRNA were analysed by qRT-PCR. (D) HUVECs were pretreated with MTX 100 nM for 54 h prior to glucose deprivation for 18 h, and measurement of apoptosis using flow-cytometric analysis of Annexin V and propidium iodide staining. (D) and (E) representative Annexin V binding histograms with pooled quantification data, and (F) and (G) propidium iodide staining and quantification. Black: HBSS alone; white: glucose deprivation; grey: MTX and glucose deprivation. Each experiment was performed three to six times. GD, glucose deprivation; H, HBSS; M, MTX; T, TNFα; UT, untreated. *p<0.05; **p<0.01.

FA is routinely prescribed alongside MTX to reduce side effects. When HUVECs were treated with clinically relevant concentrations of FA (50 nM)^27^ and MTX in combination, no change in the magnitude of MnSOD induction was observed (figure 5A).

Patients with RA and also those with primary coronary artery disease develop endothelial dysfunction as an early feature. TNFα is an important mediator and was chosen to model endothelial dysfunction in vitro. ECs were exposed to TNFα 1 ng/mL for 24 h prior to the addition of MTX for 48 h. MTX-induced upregulation of MnSOD and HO-1 mRNA was preserved (figure 5B, C). These findings confirm the ability of MTX to condition ECs in the face of a chronic proinflammatory stimulus.

### MTX protects against endothelial apoptosis induced by glucose deprivation

Next, the cytoprotective actions of MTX were investigated. The principal function of AMPK activation is to conserve energy; it is, therefore, critically important in the cellular response to glucose deprivation.^1^ ^4^ We hypothesised that survival of ECs exposed to a glucose-deficient medium would be prolonged if they were pretreated with MTX, as AMPK signalling would already be active.

ECs were treated with MTX 100 nM for 48 h, and then maintained for 18 h in Hanks’ balanced salt solution (HBSS) or glucose-deficient HBSS. Early apoptosis was detected by Annexin V staining, and established cell death by permeability to propidium iodide (PI), using flow-cytometric quantification. Glucose deprivation led to a marked increase in Annexin V binding to the EC surface and doubling of PI-positive cells. These responses were significantly reduced by MTX (figure 5D–G). Moreover, MTX did not increase EC apoptosis under normoglycemic conditions, although there was a modest reduction in total cell numbers consistent with impaired proliferation (see online supplementary figure V). These data suggest that the primary effect of MTX treatment on vascular EC is protective and that AMPK activation and subsequent protective gene induction do not represent a stress response to a noxious stimulus.

### MTX phosphorylates AMPK and increases MnSOD in murine aortic tissue and reduces endothelial and adventitial ICAM-1 expression

**Figure 6:**
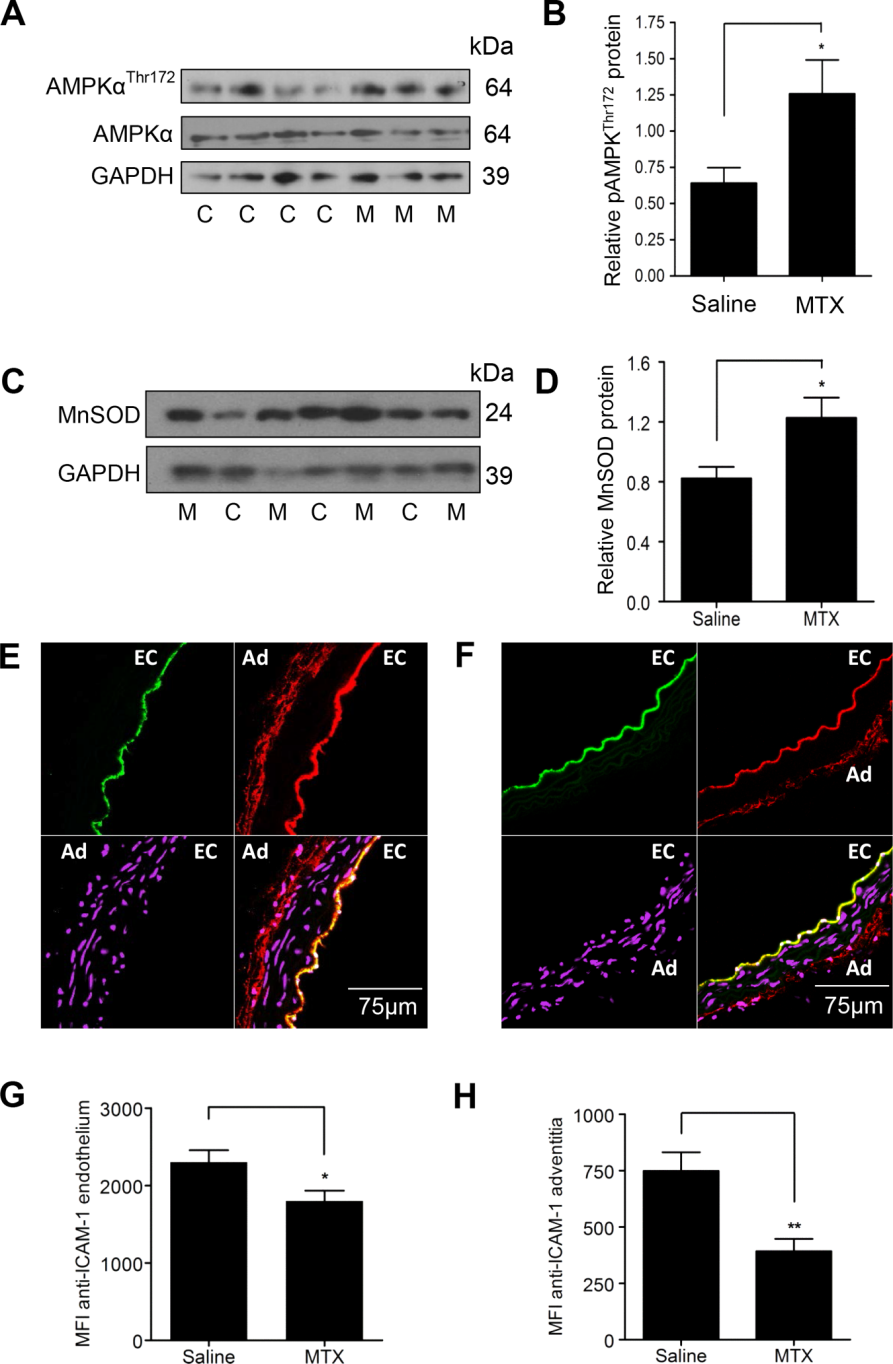
Treatment of WBF1 mice with methotrexate (MTX) leads to AMP-activated protein kinase α (AMPKα) phosphorylation and manganese superoxide dismutase (MnSOD) induction in the aorta and reduces intercellular adhesion molecule (ICAM)-1 expression. Male WBF1 mice were treated with MTX 1 mg/kg or an equal volume of 0.9% saline (n=14 in both groups) by intraperitoneal injection weekly for 4 weeks from 12 to 16 weeks of age. After euthanasia at 16 weeks, the descending aorta was snap-frozen and lysed for immunoblotting, or embedded upright in optical cutting temperature compound, and 10 μm transverse sections cut. (A) and (B) representative immunoblot and densitometry of aortic AMPKα^Thr172^ phosphorylation. (C) and (D) representative immunoblot and densitometry of aortic MnSOD. (E) and (F) representative confocal images from an animal treated with (E) 0.9% saline and (F) MTX. EC, endothelium and Ad, adventitia. Sections are stained with anti-ICAM-1 (red), anti-CD31 as an endothelial marker (green); and DRAQ5 nuclear stain (purple). (G) Quantification of endothelial ICAM-1 staining. (H) Quantification of adventitial ICAM-1 staining. Data are expressed as mean fluorescence intensity (MFI). M, individual MTX-treated animal; C, individual control animal treated with 0.9% saline; *p<0.05; **p<0.01.

Finally, snap-frozen descending aortae harvested from the MTX-treated and saline-treated animals described above were used to analyse the effect of MTX therapy on the protective pathways in vivo. Immunoblotting of WBF1 aortic lysates revealed increased AMPKα^Thr172^ phosphorylation and MnSOD protein expression in MTX-treated animals (figure 6A–D).

Given that MTX is also a powerful anti-inflammatory agent, it is likely that vascular protection induced by MTX is mediated, at least in part, by anti-inflammatory actions. To assess this, aortic intercellular adhesion molecule (ICAM)-1 expression was quantified. Transverse aortic sections from MTX and saline-treated WBF1 mice were stained with an antimouse ICAM-1 monoclonal antibody and examined by immunofluorescence confocal microscopy. MTX therapy reduced both endothelial and adventitial ICAM-1 staining (figure 6E–H). The reduction in adventitial staining is likely to be of particular relevance in the amelioration of organ damage in this model, given the reduction in the adventitial leucocytic infiltrate also observed in the intramyocardial arteries (figure 1A–C).

## Discussion

The current study identifies a novel MTX-activated protective pathway which may underpin the ability of MTX to reduce CVD associated with chronic inflammation. However, the historical general perception of MTX is that it is harmful and clinically effective only because it kills pathological tissue before normal cells are irreversibly damaged. Indeed, early studies on MTX and CVD in RA suggested CVD was increased.^28^

Subsequent experience is, however, altering perceptions. Several studies have demonstrated that long-term low-dose MTX therapy in RA (15–20 mg/week) is associated with reduced CVD, with Choi *et al* reporting a 70% reduction in cardiovascular (CV) mortality.^7^ ^8^ Likewise, MTX therapy reduced atheroma in cholesterol-fed rabbits.^29^ These results might reflect an antiatherogenic action of adenosine via ligation of its A_2A_ receptor which, in addition to an anti-inflammatory action, may induce reverse cholesterol transport proteins and prevent foam cell formation.^30^ However, our study supports an additional mechanism, namely that MTX exerts a direct beneficial effect on vascular endothelium.

Patients with RA and SLE with normal epicardial coronary arteries exhibit coronary microvascular dysfunction which may precede and contribute to accelerated atherosclerosis.^31^ Thus, to explore the arterioprotective actions of MTX, we investigated WBF1 mice, which develop an inflammatory vasculopathy of small muscular arteries and arterioles that predispose to thrombosis and tissue infarction. A previous attempt to treat WBF1 mice with MTX failed to show improvement in mortality or renal function at 30 weeks.^32^ However, the study used threefold higher doses of MTX than stated herein, and this may have contributed to mortality, given that MTX accumulates in renal failure which is universal in older WBF1 mice. Treatment of mice with early disease demonstrated that MTX reduces the severity of the vasculopathy and attenuates organ damage. MTX exerted specific anti-inflammatory actions, reducing aortic endothelial and adventitial ICAM-1 and preventing leucocytic infiltration of the arterial wall, suggesting an additional mechanism by which MTX may retard CV events in RA. However, despite the fact that MTX therapy activates protective pathways and that basic pathogenic mechanisms of arterial injury in the WBF1 mice including immunoglobulin deposition, complement activation, focal leucocyte infiltration and endothelial damage are generally applicable across many systemic inflammatory diseases, they do not necessarily translate to the situation in human RA. Thus, further studies are now required in patients.

AMPK is associated with anti-inflammatory and desirable metabolic changes in many different systems and disease settings.^1^ ^33^ Understanding of the relationship between AMPK, endothelial dysfunction and atherogenesis remains incomplete. However, current thinking suggests that reduced AMPK activity predisposes to endothelial dysfunction, while AMPK activation by laminar shear stress may contribute to vasculoprotection.^20^ Thus, our finding that MTX activates AMPK in human ECs may provide an important mechanistic explanation for the clinical observation of reduced CVD in patients with RA, prescribed this drug.

The current study demonstrates AMPK phosphorylation and induction of protective target genes using MTX concentrations in vitro that are achievable in patient sera.^22^ Moreover, increased phosphorylated AMPK and upregulation of the downstream target MnSOD were identified in murine aortae following MTX administration using a regimen analogous to the long-term, low-dose therapy used for chronic inflammatory disease. This strongly suggests that MTX-mediated AMPK activation is likely to be a real phenomenon in patients. We have also reported that, in vitro, the anti-inflammatory drug, celecoxib, can specifically induce EC AMPK phosphorylation,^34^ while at supra-therapeutic concentrations, metformin has a similar effect.^6^ Further investigation is required to establish the extent to which these observations can be directly translated to patients.

Although we have yet to determine how MTX activates AMPK, we speculate that this is secondary to increased intracellular ZMP and AMP levels. ZMP and AMP bind to the AMPKγ subunit, delaying dephosphorylation of Thr^172^ in the α subunit. Exposure of EC to MTX resulted in delayed AMPK phosphorylation, consistent with inhibition of AICAR transformylase and adenosine deaminase leading to accumulation of ZMP and AMP. These enzymes are most potently inhibited by MTX-polyglutamates, an important fact, given that MTX is rapidly converted to MTX-polyglutamates, the erythrocyte concentrations of which are more closely associated with clinical responses than MTX plasma levels.^11^ MTX is known to increase AICAR levels in HUVECs after a 48 h treatment^12^ and can enhance activation of AMPK by AICAR in cancer cell lines.^35^ While this manuscript was under review, Pirkmajer et al have shown that MTX and AICAR together increase ZMP in cultured myotubes, supporting our hypothesis as to how MTX activates AMPK.^36^

Previous studies have shown that the AMPKα1 isoform is more abundant in ECs and have, therefore, focused on its functional effects.^6^ ^23^ Recent evidence suggests that important vasculoprotective effects are also mediated through AMPKα2.^5^ Our study demonstrates that depletion of either α subunit attenuates MTX-mediated MnSOD and HO-1 induction. For the regulation of MnSOD, AMPKα2 activity seemed somewhat more important than α1, a fact supported by the finding that addition of SOD to aortae from AMPKα2^−/−^ mice can rescue endothelial dysfunction.^5^ Interestingly, the α2 subunit is thought to be the more sensitive to AMP,^37^ and this is consistent with our speculation that MTX activates AMPK by altering levels of AMP.

MTX treatment attenuated apoptosis induced by glucose deprivation, a response replicated by the AMPK agonist AICAR.^4^ The underlying mechanism is likely multifactorial. AMPK activation moves cells away from glycolysis as an ATP source, and promotes mitochondrial oxidative phosphorylation, thus prolonging cell survival in low glucose conditions. Additionally, induction of A1 and HO-1 by MTX may exert antiapoptotic effects. MnSOD, UCP-2 and HO-1 induction will also impart important antioxidant effects which improve cell survival. Although beyond the scope of this initial report, induction of additional AMPK targets A1, UCP-2 and DAF suggest that a whole-genome approach is now indicated to identify the full extent of the cytoprotective profile of MTX and to determine how its actions may be replicated by novel, more specific compounds.

The demonstration that MTX activates an AMPK/CREB-dependent signalling pathway to induce MnSOD and HO-1 reveals significant atheroprotective potential. MnSOD induction protects against atherogenesis, and ApoE^−/−^ mice deficient in MnSOD exhibit accelerated atherosclerosis.^21^ Recent evidence points towards a central role for CREB in the maintenance of a healthy arterial wall. Loss of aortic CREB is found in rodent models of hypertension, atherosclerosis and insulin resistance,^25^ while murine cardiac-specific expression of dominant-negative CREB increases oxidative stress, mitochondrial dysfunction and mortality.^38^ Although CREB^Ser133^ is a direct target of AMPK,^24^ CREB activation by MTX might also occur via adenosine binding of G protein-coupled receptors, leading to protein kinase A activation via cAMP. However, this well-described mechanism of CREB activation^39^ is thought only to occur on promoters where the CRE site is within 250 bp of the TATA box.^40^ The SOD2 promoter does not contain a TATA box,^41^ and the CRE site likely to be responsible for the effects of CREB binding presented here lies 1200 bp distal to the transcription start site. This favours AMPK activation rather than adenosine as the route to CREB activation by MTX.

The lack of evidence demonstrating a requirement for AMPK-CREB signalling for the protective effects of MTX in vivo is a limitation of this study. Their complex genetic background and the need to generate F_1_ animals to develop vasculopathy precludes crossing WBF1 mice with an AMPK-deficient strain. Likewise, data obtained by treating animals daily with the AMPK antagonist, Compound C, would be confounded by the observation that the drug itself induces protective enzymes including HO-1 and MnSOD.^42^ However, AMPKα activation and MnSOD induction were demonstrated in aortae from MTX-treated animals, suggesting they play a role in vascular protection. Nevertheless, increased adenosine may also contribute to the vascular effects of MTX. Adenosine reduces cytokine-mediated cell adhesion molecule upregulation in EC,^43^ and MTX treatment promotes vasodilatory responses attributed to ligation of adenosine 2A receptors.^44^

In conclusion, we have presented evidence for a novel MTX mechanism of action in the vasculature, which identifies specific effects of low-dose MTX and moves the perception of this drug from toxic to protective. We propose that low-dose MTX therapeutically conditions vascular endothelium via activation of AMPK-CREB signalling, so inducing cytoprotective genes which may contribute to the efficacy of MTX in reducing CV complications in patients with RA.

## Supplementary Material

Web supplement
