# Supplementary material for: Methotrexate-mediated activation of an AMPK-CREB-dependent pathway: a novel mechanism for vascular protection in chronic systemic inflammation
Source: Ann Rheum Dis. 2014 Dec 30;75(2):439–48. doi: 10.1136/annrheumdis-2014-206305 (PMC4752671; doi:10.1136/annrheumdis-2014-206305)
Supplement: Web supplement [file annrheumdis-2014-206305-s1.pdf]

Supplementary Data File

Methods

In vivo analysis

All animals were studied according to UK Home Office guidelines and with ethical approval from Imperial College London (Licence PPL 70/6722). All experiments were performed in accordance with the principles of reduction, replacement and refinement. Mice were housed under controlled climatic conditions in microisolator cages with autoclaved bedding, a 12h light/dark cycle and unlimited access to food and water. NZW females (Harlan, UK) were crossed with BXSB males (bred in-house) and the male offspring (WBF1) were used as the study population. Alternate animals were allocated to receive MTX (n=19) or 0.9% saline control (n=18), thus ensuring littermate controls were used, and a coding identification system facilitated blinding of the investigators. From 12-16 weeks of age, mice were treated weekly with MTX 1mg/kg (TEVA, Castleford, UK) or an equal volume of 0.9% saline by intra-peritoneal injection. Animals were monitored daily for signs of ill-health. The dose of MTX chosen was based on two previous studies in which 0.5mg/kg/week or 2.5 mg/kg/week were used and shown to increase adenosine levels and protect against collagen-induced arthritis.[1 2] A mid-range dose of 1 mg/kg/week was chosen for this study to maximise the chances of seeing an effect while minimising the effect of toxicity given the propensity of WBF1 mice to develop renal impairment.

After euthanasia, the heart was fixed in 2% buffered neutral formalin (Sigma-Aldrich) for 24h, then bisected at a mid-ventricular point and paraffin-embedded. The descending aorta was placed on ice in physiological saline. Excess fat and connective tissue was trimmed by microscope dissection, and the aorta was divided in two transversely. One half was embedded upright in OCT compound (Cell Path, Newtown,UK) and snap frozen in supercooled methylbutane (Sigma-Aldrich), for frozen sectioning. The other half was snap frozen in liquid nitrogen, then ground using a pestle before lysis in RIPA buffer for immunoblotting.

Quantification of myocardial infarct area

5µm sections of WBF1 hearts were stained using a Picrosirius Red kit (Polysciences Inc, Warrington, PA). Identification of myocardial infarcts was confirmed by two investigators. Consecutive photomicrographs of the area of infarct were taken using an Olympus BX50 microscope, and a single

image of the whole section was generated using a JVC KY-F1030V digital camera. The area of fibrotic infarct was calculated by thresholding using Image J software to analyse the area of red pixels representing collagen deposition. In addition, acute infarct area was quantified using Image J and expressed as a percentage of the whole section. The two values were added together to give total infarct area as a proportion of myocardial section area.

Quantification of vasculopathy

Cardiac sections stained with periodic acid-Schiff (PAS) were examined blind by two investigators. A modified scoring system for the vasculopathy of the intramyocardial arteries and arterioles based on Berden et al[3] was constructed. The peri-adventitial leukocytic infiltrate was quantified by counting the number of cells in the adventitia of each intramyocardial artery and arteriole in one section for each animal. Deposits of PAS-positive material within the arterial wall were scored as follows: 0 = normal staining, 1 = increased staining of the basement membrane, 2 = increased basement membrane staining and presence of nodular deposits.

Aortic immunofluorescence

Transverse frozen sections of murine aorta were blocked in 3% BSA/PBS, then incubated with rat anti-mouse ICAM-1 monoclonal antibody YN-1 (American Type Culture Collection, Manassas, VA) or rat IgG_{2a} isotype control (BD Pharmingen), followed by an Alexa Fluor 546-conjugated goat anti-rat IgG secondary antibody (Invitrogen, Carlsbad, CA). A directly conjugated Alexa Fluor 488 rat anti-mouse CD31 antibody (MEC13.3; Biolegend, San Diego, CA) was used as an endothelial marker, and DRAQ5 (Biostatus, Loughborough, UK) as a nuclear stain. Slides were mounted with Fluoromount-G (Southern Biotech, Birmingham, AL) and visualized and quantified by confocal microscopy (LSM 510 Meta, Carl Zeiss, Oberkochen, Germany). Scan settings were set to optimize the signal/noise ratio for each emission wavelength and there was no detectable crossover between channels. The endothelial and adventitial areas were first identified using the anti-CD31 stained co-localized image and then, to measure ICAM-1, the mean fluorescence intensity of the area in the red channel (corresponding to AlexaFluor 546 staining) was recorded. Processing and quantification was performed with the Zeiss LSM Image Browser using the histogram function.

Endothelial Cell Culture

The study of human umbilical vein (HUVEC) and human arterial endothelial cells (HAEC; Promocell, Heidelberg, Germany) was carried out as previously described [4]. HAEC were studied at passage 7-8 and HUVEC at passage 2-4 in experimental medium consisting of M199 (Sigma-Aldrich, St Louis, MO) supplemented with 10% fetal bovine serum (Biosera, Boussens, France), 15µg/ml endothelial cell growth factor (Sigma-Aldrich), 10 units/ml heparin, 100µg/ml streptomycin/ penicillin (Gibco Life Technologies, Carlsbad, CA) and 2mM L-glutamate (Gibco). EC were treated with MTX (Tocris Bioscience, Bristol, UK), AICAR (Calbiochem, Merck Chemicals Ltd, Nottingham, UK), folic acid (Sigma-Aldrich), TNFα (R&D Systems, Minneapolis, MN) and Compound C (Calbiochem).

Western Blotting

HUVEC or murine aortic tissue lysates were prepared in RIPA buffer (Thermoscientific, Waltham, MA) supplemented with complete protease and phosphatase inhibitors (Roche Diagnostics Ltd, West Sussex, UK). Following quantification of protein concentration by the Bradford assay (Bio-Rad, Hercules, CA), 10-20µg protein was loaded into 4-12% gels (Invitrogen, Carlsbad, CA) and run at 150V for 1.5h, followed by semi-dry transfer to PVDF membrane at 0.1A per membrane for 1-2h. Membranes were blocked for 1h and incubated with the primary antibody at 4°C overnight. Following incubation with an appropriate HRP-conjugated secondary antibody and development with ECL chemiluminescence (GE Healthcare, Little Chalfont, UK), films were scanned using a Canoscan (Canon, Tokyo, Japan) and pixel density of bands quantified in Image J (National Institute of Health, USA) (antibodies are listed in Table 1).

**Target Antigen**	**Species**	**Supplier**	**Concentration**
AMPKα	Rabbit	Cell Signaling	1:1000
phospho AMPKα^{Thr172}	Rabbit polyclonal	Abcam	1:1000
CREB-1	Rabbit	Cell Signaling	1:500
phospho CREB-1^{Ser133}	Rabbit	Cell Signaling	1:1000
GAPDH	Mouse monoclonal	Millipore	1:25000
HO-1 (OSA-111)	Mouse monoclonal	Abcam	1:2000
MnSOD	Rabbit monoclonal	Assay Designs	1:2000
α-Tubulin	Mouse monoclonal	Sigma-Aldrich	1:25000
HRP-tagged anti-mouse IgG	Goat	DAKO	1:3000 – 1:30000
HRP-tagged anti-rabbit IgG	Swine	DAKO	1:3000 – 1:30000

Table 1. Suppliers and concentrations of primary and secondary antibodies used in Western blots.

Quantitative real-time RT-PCR

RNA extraction from HUVEC was carried out using an RNEasy mini kit (Qiagen, Venlo, Limburg, Netherlands) including incubation with DNAse (Qiagen), and RNA concentration was measured using a Nanodrop 2000 (Thermoscientific). cDNA was synthesised using Q-script supermix (Quanta Biosciences, Gaithersburg, MD) on a T3 Thermocycler (Biometra GmBH, Göttingen, Germany). The real-time PCR was carried out in triplicate using a CFX96 Real-Time System C1000 Thermal Cycler (Bio-Rad). Glyceraldehyde-3-phosphate dehydrogenase (GAPDH) and β-actin were used as housekeeping genes. Primer sequences are shown in Table 2. Relative mRNA abundances were calculated using the comparative cycle threshold method.

**Target**	**Sequences**
A1	Forward 5’-CAC AGG AGA ATG GAT AAG GCA AA-3’<br>Reverse 5’-AGT CAT CCA GCC AGA TTT AGG TTC-3’
AMPKα1	Forward 5’-TCA GGA AGA TTG TAT GCA GGC CCA-3’<br>Reverse 5’-TTC ATG GGA ATC CAC CTG CAG GAT TA-3’
AMPKα2	Forward 5’-CGA GCT ATG AAG CAG CTG GA-3’<br>Reverse 5’-GAA CGC TGA GGT GTT GAG GA-3’
β-Actin	Forward 5’-GAG CTA CGA GCT GCC TGA CG-3’<br>Reverse 5’-GTA GTT TCG TGG ATG CCA CAG GAC T-3’
DAF	Forward 5’-CCT TAA GGG CAG TCA ATG GT-3’<br>Reverse 5’-CGG CAC TCA TAT TCC ACA AC-3’
GAPDH	Forward 5’-CAA CAG CCT CAA GAT CAT C-3’<br>Reverse 5’-GAG TCC TTC CAC GAT ACC-3’
HO-1	Forward 5’-CAG TCT TCG CCC CTG TCT AC-3’<br>Reverse 5’-CTG GTG TGT AGG GGA TGA CC-3’
MnSOD	Forward 5’-AAG GGA GAT GTT ACA GCC CAC-3’<br>Reverse 5’-GTC CAG AAA ATG CTA TGA TTG A-3’
UCP-2	Forward 5’-GGA TAC TGC TAA AGT CCG GT-3’<br>Reverse 5’-CCA TTG TAG AGG CTT CGG G-3’

Table 2. Primer sequences used for quantitative real-time RT-PCR.

RNA Interference

Transfection of HUVEC with short interfering RNA (siRNA) was performed using GeneFECTOR lipid transfection reagent (VennNOVA, Pompano Beach, FL). HUVEC were incubated in Optimem serum-free medium (Gibco) for 6h, along with scrambled control (Dharmacon, Epsom, UK) or specific siRNA (Qiagen) complexed with GeneFECTOR (sequences are available in Table 3). Following recovery in EGM-2 medium for 18h (Lonza, Basel, Switzerland), EC were washed and incubated in experimental medium for stimulation with MTX.

**Target gene**	**siRNA name**	**mRNA target sequence 5'-3'**	**Company**
AMPKα1	Hs_PRKAA1_5	CCC ACG ATA TTC TGT ACA CAA	Qiagen
AMPKα2	Hs_PRKAA2_6	CCG AAG TCA GAG CAA ACC GTA	Qiagen
CREB-1	CREB-1 pooled sequences siGENOME SMARTpool	GAG AGA GGT CCG TCT AAT G<br>CGT ACA AAC ATA CCA GAT T<br>GAG TGG AGA TGC AGC TGT A<br>TGA CTT ATC TTC TGA TGC A	Dharmacon

Table 3. siRNA sequences used for RNA interference.

Induction of apoptosis and detection by flow cytometry

Apoptosis of human umbilical vein endothelial cells (HUVEC) was induced by incubation in glucose-free Hanks' balanced salt solution (HBSS), supplemented with 2% bovine specific albumin (Sigma-Aldrich, St Louis, MO) and 2mM L-glutamate (Gibco Life Technologies, Carlsbad, CA), for 18h. HUVEC were trypsinized and centrifuged at 1200 rpm for 5 minutes, then resuspended in Annexin V binding buffer (BD Pharmingen, San Diego, CA). Following incubation with PerCP-Cy5.5-Annexin V (BD Pharmingen) for 30 minutes and propidium iodide (Sigma-Aldrich) for 15 minutes at 4°C, staining was analysed by counting 10,000 cells in a Beckman-Coulter CyAn flow cytometer.

Chromatin immunoprecipitation

Chromatin immunoprecipitation (ChIP) was performed using the ChIP-IT Express kit (Active Motif, Carlsbad, CA) as previously described[5]. HUVEC stimulated for 48h with MTX were fixed for 5 minutes in 1% formaldehyde, then detached by scraping. Following nuclear lysis, chromatin was sheared by sonication at 4°C for 4 minutes in 30s pulses using a Bioruptor (Diagenode, Denville, NJ). Immunoprecipitation was performed at 4°C overnight using 2µg anti-CREB-1 antibody or IgG control (Cell Signaling Technologies, Danvers, MA). Following retrieval of chromatin, quantitative real-time PCR was performed using primers designed to isolate the two predicted CREB binding sites in the manganese superoxide dismutase (MnSOD; SOD2) promoter (MatInspector by Genomatix), and a negative control downstream site 2kbp from the transcription start (see Table 4 for sequences).

Results were analyzed using the comparative cycle threshold method, normalizing to input and IgG control for each condition.

Primer Site	Sequences
1	Forward 5’-TGC AAA TCC TGC CTG CAG TCT C-3’<br>Reverse 5’-GCG CCG TAC CCT TGC TTT GC-3’
2	Forward 5’-GTG CCA GAC CAC CTT GCC TGA-3’<br>Reverse 5’-AAC GCA GAC AAG AGC AGG GGT-3’
Downstream	Forward 5’-GGC ACA CCG TTG TGG TGT CGT-3’<br>Reverse 5’-AAC AAG CCT GTT TCA GTT TCC CAC T-3’

Table 4. ChIP primer sequences.

8. Statistical Analyses

All data were analyzed using GraphPad Prism 4 (GraphPad Software, La Jolla, CA). Numerical data are presented as mean and standard error. Results were tested for normality using a d'Agostino and Pearson omnibus normality test and data that passed this test were analysed using paired or unpaired Student's t-tests or one-way ANOVA as appropriate. Normalized data were analysed using the single sample t-test. Data that were not normally distributed were analyzed using a Mann-Whitney test. Differences were considered significant at $p<0.05$.

References

1. Al-Abd AM, Inglis JJ, Nofal SM, et al. Nimesulide improves the disease modifying anti-rheumatic profile of methotrexate in mice with collagen-induced arthritis. *Eur J Pharmacol*. 2010;644:245-50.
2. Cronstein BN, Naime D, Ostad E. The antiinflammatory mechanism of methotrexate. Increased adenosine release at inflamed sites diminishes leukocyte accumulation in an in vivo model of inflammation. *J Clin Invest*. 1993;92:2675-82.
3. Berden JH, Hang L, McConahey PJ, et al. Analysis of vascular lesions in murine SLE. I. Association with serologic abnormalities. J Immunol. 1983;130:1699-705.
4. Mason JC, Ahmed Z, Mankoff R, et al. Statin-induced expression of decay-accelerating factor protects vascular endothelium against complement-mediated injury. Circ Res. 2002;91:696-703.
5. Dryden NH, Sperone A, Martin-Almedina S, et al. The transcription factor Erg controls endothelial cell quiescence by repressing activity of nuclear factor (NF)-kappaB p65. *J Biol Chem*. 2012;287:12331-42.

Supplementary Figures

Feature	Score
Death	2
Myocardial infarct: full thickness	2
Myocardial infarct: small epicardial	1
Bowel infarct	1
Aortic aneurysm	1
Ascites or pleural fluid	1
Hepatosplenomegaly	1

Supplementary Data Table

Post-mortem findings used to construct the WBF1 organ damage score.

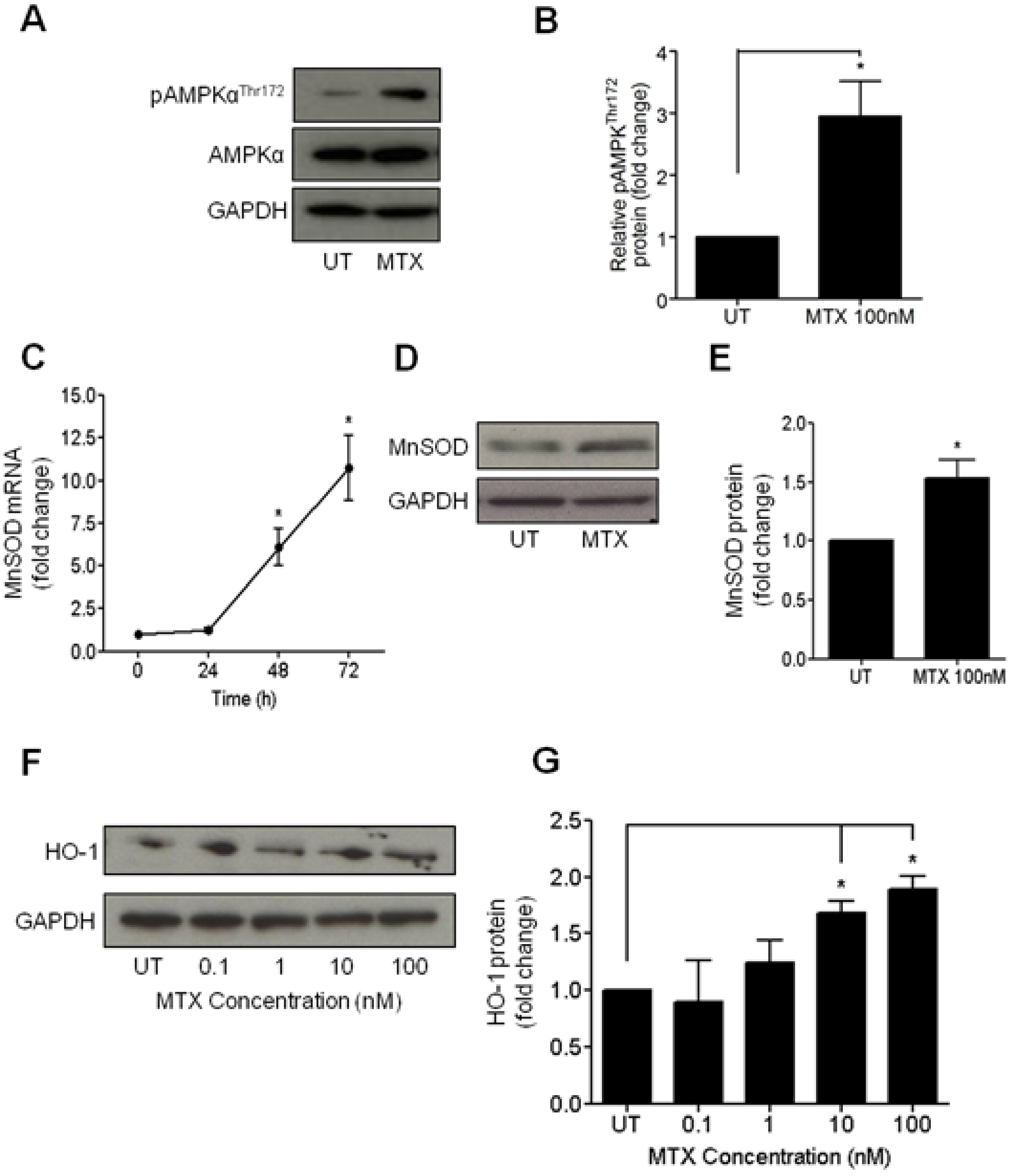

Supplementary Figure I

A: Representative immunoblot of $AMPK\alpha^{Thr172}$ phosphorylation in HAEC by MTX (100nM for 48h).

B: Densitometry demonstrating $AMPK\alpha^{Thr172}$ phosphorylation in HAEC by MTX (100nM for 48h).

C: Time course of MnSOD RNA induction in HUVEC by MTX (100nM).

D: Representative immunoblot of MnSOD protein induction in HAEC by MTX (100nM for 48h).

E: Densitometry demonstrating induction of MnSOD protein in HAEC by MTX (100nM for 48h).

F: Representative immunoblot of HO-1 protein induction by MTX for 48h in HUVEC.

G: Densitometry demonstrating HO-1 protein induction by MTX for 48h in HUVEC.

*p<0.05.

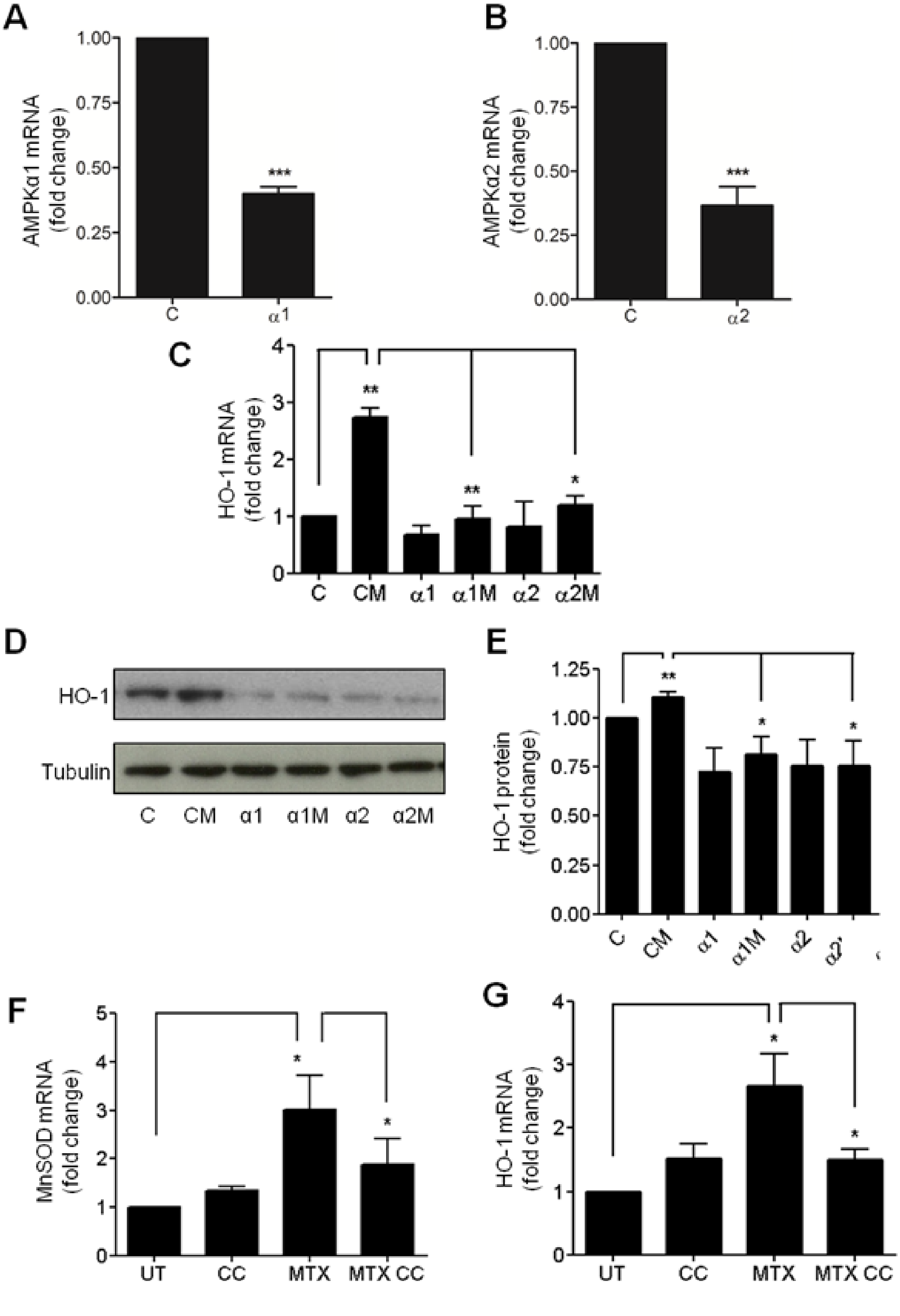

A
AMPKα1 mRNA (fold change)
1.00
0.75
0.50
0.25
0.00
***
C
α1
B
AMPKα2 mRNA (fold change)
***
α2
C
HO-1 mRNA (fold change)
**
**
*
C CM α1 α1M α2 α2M
D
HO-1
Tubulin
C CM α1 α1M α2 α2M
E
HO-1 protein (fold change)
1.25
1.00
0.75
0.50
0.25
0.00
F
MnSOD mRNA (fold change)
UT CC MTX MTX CC
G
HO-1 mRNA (fold change)
UT CC MTX MTX CC

Supplementary Figure II

A: Efficiency of knockdown of AMPKα1 subunit mRNA with siRNA

B: Efficiency of knockdown of AMPKα2 subunit mRNA with siRNA

C: HUVEC treated with control (C), AMPKα1 or AMPKα2 siRNA and exposed to MTX (100nM for 48h). HO-1 mRNA was quantified by qRT-PCR.

D-E: HUVEC were treated with control (C), AMPKα1 or AMPKα2 siRNA and exposed to MTX (100nM for 48h) and HO-1 HO-1 protein analyzed by immunoblotting.

F-G: HUVEC were pre-treated with vehicle alone or Compound C prior to addition of MTX (100nM) and qRT-PCR analysis of (F) MnSOD and (G) HO-1 mRNA by MTX 100nM.

Abbreviations: α1: AMPKα1 siRNA; α2: AMPKα2 siRNA; C: scramble control siRNA; M: MTX 100nM for 48h; OD: optical density; CC: Compound C; UT: untreated. *= $p<0.05$; **= $p<0.01$; ***= $p<0.001$.

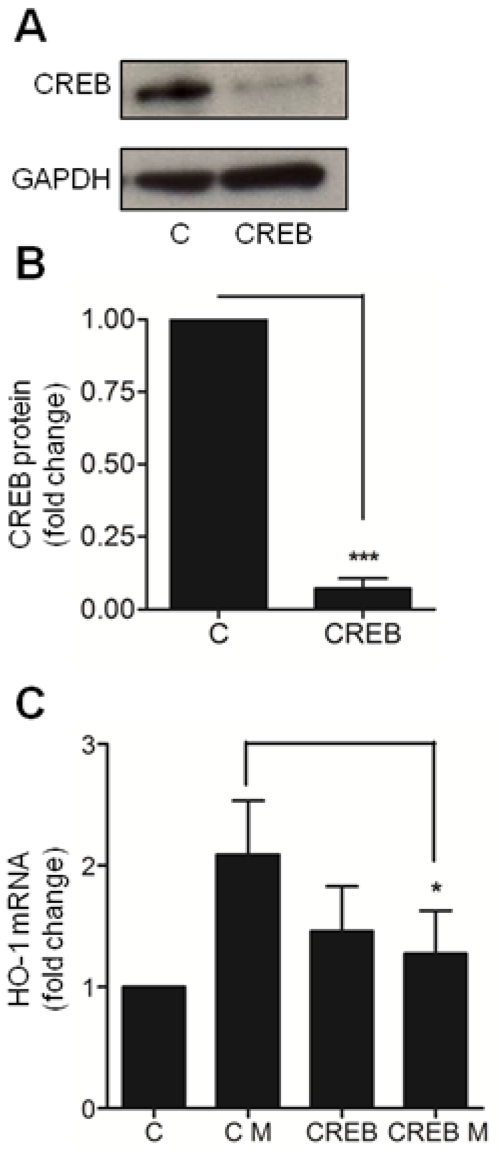

Supplementary Figure III

A-B: HUVEC were treated with control (C) or CREB siRNA (CREB) prior to immunoblotting CREB and quantification by densitometry.

C: HUVEC were treated with control (C) or CREB siRNA (CREB) prior to exposure to MTX 100nM for 48h and analysis of HO-1 mRNA by qRT-PCR.

*= $p<0.05$; **= $p<0.01$; ***= $p<0.001$.

```
1    ggatccttac aatggagata gtggggccag gcatggtggc tcatgcctgt aatcccagca  ctttgggagg ctgaggcagg cagatcactt cagtctagga
101  gttcgagacc agcctggcca acatggtgaa accccatctc cactaaaaat acaaaaatta gccaggcatg gtggcacgca  cctgtaatcc cagctactca
201  ggaggctaag gcaggagaat cacttgaact caggaggtgg aggttgcagt gagccgagat cgcaccactg cactccagcc cagcaacaaa gcaagactct
301  tgattcggaa agaaaaaata aaaaaaggtt gggggagaca gtgggagccc agacttttgt ccttcccctt gcctagaagg gagatgaggt tgctggtgct
401  gtggaagcta ttatggacca  tgaggcagct ttgaagacag aaagcctgca tccttgatga ctcaatggaa tttcatccca acccaggact gactgcccca
501  gatctttgtt acatgaggga atacactctt ctgtgtttaa  gaaactgtgg ttggatctgt tactggaata tgaatgcagt taataactgg ctcaaaatga
601  cttcatctca acccacattg gttgcttctt ccaaacaata tataggtctg tactgtggtg  ggtctcagga tggctgtgat gtagccttag gaagtttatc
701  tatgggaaat ccatattcat ggtgtcctga tgttgcagag gacatcctga gctggctgga gtaacttggg acacaggtca   atcgactgta atctaacttc
801  tgaggccatt cagtaccctc tacagtggcc acctaaaaaa aaggcagcca ggtgtggtgg ctcaagccta tatagatccc agaactttgg aaggctgagg
901  tgggaggatc acttgagcca aggagtttga ggccagcctg ggcaacatga agaaactctg tttctacaaa aaataaaaaa aattagccag gcatggtggt
1001 atgcacctgt agttccagct tcttgggagg ctgaggtgga agaatgacat gagcccagca agtcgctgca atcagccgtg atcacgccgc tgcactccag
1101 cctgggcgac aaaaagaaaa agaaaacgga gcctgttcac tgggtgtggt agacaaggta aacttttctt tacctcccat atcccacaac cttggatgtg
1201 ctcacagtca tggtagtgtt ttgaatgatg tagctgatga caggtgtgat gttggagatt ctctacctga ctgctgctat cagtctacca gcccccaacg
1301 tttggtgctt gttctaaagg gcatgtccta ggagtcgctt taaagagctc agcctcccaa taggaatatt tcattatcac tagatcaagt ctttccatta
1401 caatgactga tctgtctctg aatcctgtgg attcatcctt caaaatgccc ttttcttcca gttctcatag ctagtgccct taaaagtgac ctgcagtacc
1501 tcctgctgag acgaatgtac cagcttccta actagcctgc actcccttca tccccccaag tcagtgccag accaccttgc ctgaaaaacc actttcagtg
1601 tgtctcacct cagcagaaat gtttctcagc ttccaattaa caatcacatc aaacccctgc tcttgtctgc gttttaaggg tatctatagg ccgggcgccg
1701 tggctcctac ctgtaatccc agcactttgg aaggccgagg cgggcagatc actTGACGTC Aggcgttcga gaccatcctg accaacatag tgaaaccccg
1801 tctctaccaa aaatacaaaa aaaaaaaaaa tagtgggcgt ggaggtgcac gcctgtaatt ccagctactc gggaggctga ggcaggagaa tcgcttgaac
1901 ccgggaggca gaggttccag tgagccgaca tcgcgacaca gtactccagc ctgagcgaca gagcgaggct gtgtctcaaa aataaataaa taataaattt
2001 aaaaaaataa gagtatctat aacctggtcc cagcctgaat ttcctttttc acccccaaca cgtagcccta gttacattct tcTGACGTct gtaaacaagc
2101 ccagcccttc ctgttgtgaa gccaagttca ggtggttcct cttcgcctga ctgttttccc attccactta ccggaagcct agtcatcctt cggagggctg
2201 tacgggggtt gcaagaagca acggaaacgg ttcagcacct gctaccttcc atcatattct tttcaataaa ggggcaactc ccgcaatggc agtgtagatt
2301 tcctaacctc tacacatgga agattcacac cattcaggat tgttgtttaa ctgttgagag agcacttgat acttaacagc ttactaggct acaagacagc
2401 gcaggaaaga atcctctgtt gtccttttat gttatcctga acagttggtt cacagagtta ctgtaaacac acaaaacatg actgccaggg cttagtagtg
2501 aggaaggtgg gaactagtcc tgactcagtt aactgtgccc aggagaagct gcttaacctc aaaggatttc actattacta gaatcaataa taccaaccct
2601 aggggtaaaa ataaagataa atgtgtgcaa atcctgcctg cagtctcggg cacgtcgtgg gtgtccaaga actgttctta ggcagccggt ggggacaaag
2701 tctgtgtgcc tcctgtcctg gaataggtcc caaggtcggc ttacttgcaa agcaagggta cggcgcaaga gtactgaata cgggttggaa gggcgctggc
2801 tctaccctca gctcataggc cggctgggcg gcgctgacca gcagctaggc cccgtcttcc ctaggaacgg ccacgggggc cctgggaggg tatgaatgtc
2901 tttttgcagt gaggcctctg gaccccgcgg ccccccggca gcgcaaccaa aactcagggg caggcgccgc agccgcctag tgcagccaga tccccgccgg
3001 caccctcagg ggcggacgga ggagggcctt cggccgtacc aactccacgg gggcaggggc cgcctccctt cggccgcgcg ccactcaagt acggcagaca
3101 ggcagcgagg ttgccgaggc cgaggctagc ctgcagcctc ctttctcccg tgccctgggg cggtgtacgg caagcgcggg cgggcgggac aggcacgcag
3201 ggcacccccg gggttgggcg cggcgggcgg ggcggggccg cggggggagg gggcggggcg gcggtgccct tgcggcgcag ctggggtcgc ggccctgctc
3301 cccgcgcttt cttaaggccc gcgggcggcg caggagcggc actcgtggct gtggtggctt cggcagcggc ttcagcagat cggcggcatc agcggtagca
3401 ccagcactag cagcatgttg  agccgggcag tgtgcgggtg agaagaaagg ggacccggtc acggccccaa ggggcaaggg gctcgcggcg ggcagggcct
3501 ccgcggcaat ggcgacagtg gccgcaccgg gcctggcggg accggggcac ctgcaggcgg ttctcccggg agtgcccggc gcggcggctg gagcggggat
```

Supplementary Figure IV

SOD2 promoter (GenBank accession number AF059197), showing CREB-1 binding sites predicted by MATinspector software in red, and the transcription start site in blue.

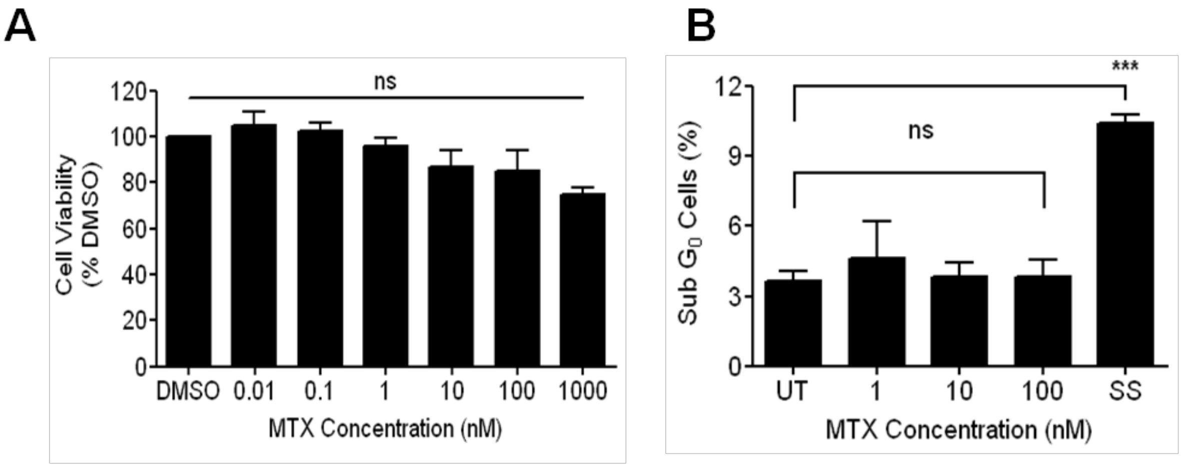

Supplementary Figure V

A: MTS assay in HUVEC treated with MTX (0-1000nM) for 48h.

B: Propidium iodide cell cycle analysis in HUVEC treated with MTX (0-100nM) or exposed to serum starvation (SS) for 48h. ***= $p<0.001$.
